# Surgical outcomes following encephaloduroarteriosynangiosis in moyamoya disease associated with hyperhomocysteinemia

**DOI:** 10.1002/brb3.3093

**Published:** 2023-06-29

**Authors:** Gan Gao, Fang‐bin Hao, Qian‐Nan Wang, Xiao‐Peng Wang, Si‐meng Liu, Min‐jie Wang, Qing‐bao Guo, Jing‐jie Li, Xiang‐Yang Bao, Cong Han, Lian Duan

**Affiliations:** ^1^ Chinese PLA Medical School Beijing China; ^2^ Department of Neurosurgery Chinese PLA General Hospital Beijing China

**Keywords:** collateral circulation, encephaloduroarteriosynangiosis, hyperhomocysteinemia, moyamoya disease

## Abstract

**Introduction:**

This study investigated the effect of indirect revascularization surgery in adult patients with moyamoya disease (MMD) complicated with hyperhomocysteinemia (HHcy), and the effect of HHcy on the progression of adult MMD.

**Methods:**

A retrospective case‐control study was conducted in patients with MMD, with or without HHcy (*n* = 123). Postoperative collateral angiogenesis was evaluated using the Matsushima grading system and disease progression using the Suzuki staging system. Cerebral blood flow was evaluated before and after surgery using dynamic susceptibility contrast magnetic resonance imaging (DSC‐MRI) and neurological function prognosis using the improved Rankin score (mRS). Univariate and multivariate logistic regression analyses were performed to determine risk factors for the clinical outcomes.

**Results:**

There was no significant difference in the Suzuki stage composition ratios between the HHcy group and the non‐HHcy group before and after surgery. Non‐HHcy patients were more likely to grow new collateral circulating vessels after encephaloduroarteriosynangiosis (EDAS). Moreover, postoperative DSC‐MRI indicated that the time to peak significantly improved.

**Conclusions:**

HHcy level may be a specific predictor of adverse clinical outcomes after EDAS in patients with MMD and a risk factor for poor collateral circulation and poor prognosis. Patients with MMD complicated with HHcy need to strictly control homocysteine levels before EDAS surgery.

## Graphical abstract

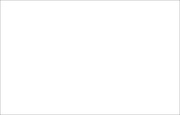

## 1 INTRODUCTION

Moyamoya disease (MMD) is a cerebrovascular disease characterized by slow and progressive thickening of the intima of the bilateral internal carotid arteries and the anterior and middle cerebral arteries, gradual narrowing or occlusion of the arterial lumen, and compensatory dilation of the basal penetrating artery (Suzuki & Takaku, 1969). Globally, the prevalence of MMD is high in China, Japan, South Korea, and other Asia‐Pacific regions (Kim et al., 2016). The highest prevalence has been reported in Hokkaido, Japan (10/100,000) (Baba et al., 2008). Recent studies have shown that intracranial and extracranial vascular reconstructions are the only effective direct methods for treating MMD (Ihara et al., 2022). At the same time, encephaloduroarteriosynangiosis (EDAS) is an indirect vascular reconstructive surgery that has proven to be effective in improving cerebral blood flow and reducing the risk of secondary stroke in children and adults with MMD (Agarwalla et al., 2014; Bao et al., 2015; Bao et al., 2012; Duan et al., 2012; Dusick et al., 2011; Ishikawa et al., 1997). Previous studies have shown that the postoperative revascularization effect of EDAS is related to age and blood glucose levels (Ren et al., 2016). However, few studies have evaluated the effect of hyperhomocysteinemia (HHcy) on postoperative revascularization and clinical outcomes in patients with MMD.

Homocysteine (Hcy) is a sulfur‐containing amino acid produced as an intermediary in methionine metabolism. Hcy concentrations are affected by dietary intake of folic acid and B vitamins, lifestyle factors such as smoking, and genetic factors (Chaabane et al., 2018; Trinh et al., 2002; Weisberg et al., 2001). Several common functional polymorphisms of genes encoding enzymes that act in the folate/Hcy metabolic pathway, such as methylenetetrahydrofolate reductase (MTHFR), are likely to contribute to variations in Hcy levels (Duan et al., 2018; Frosst et al., 1995; Lemarié et al., 2011). An increase in plasma Hcy levels results in HHcy, a condition that is an independent risk factor for the development of cardiovascular, cerebrovascular, and cerebral small vessel disease (Ganguly & SF, 2015; Hoque et al., 2008; Yuan et al., 2020). However, the effects of HHcy on cerebral neovascularization and disease progression in patients with MMD remain unclear. In this study, we investigated the differences in collateral circulation and disease progression after EDAS between patients with MMD with and without HHcy.

## 2 MATERIAL AND METHODS

### 2.1 Patient selection

A consecutive surgical series of patients with MMD who underwent EDAS between January and December 2017 at the Department of Neurosurgery, Fifth Medical Center of the Chinese People's Liberation Army, Beijing in China, was reviewed to identify all patients who were ≥18 years at the time of surgery. To avoid selection bias, we selected patients who received surgery in the year 2017 as study subjects. To eliminate the influence of contralateral cerebral hemisphere self‐compensation on collateral compensation after EDAS, we only included patients with bilateral lesions. All patients were diagnosed with MMD based on cerebral digital subtraction angiography findings. The inclusion and exclusion criteria were set in accordance with the criteria for the diagnosis and treatment of MMD (Fujimura et al., 2022). We divided all patients with MMD into two groups: patients with HHcy and patients without HHcy. All patients with HHcy had received a definite diagnosis of HHcy according to the World Health Organization's definition, namely, an increase in serum Hcy exceeding 15 μmol/L (Holmes et al., 2011). Fifty‐five patients with MMD complicated with HHcy fulfilled the inclusion criteria, and 14 were excluded due to incomplete imaging data or loss to follow‐up. Finally, 41 patients with MMD complicated with HHcy were included in the study population. Using a matched‐pair case‐control study design, 82 control participants were randomly selected from the patients with MMD without HHcy and matched according to age and sex.

### 2.2 Retrospective chart review

The patients’ clinical records, including hospital charts, clinical notes, and radiological studies, were reviewed. All data were collected in October 2022. This study was approved by the Research Ethics Committee of the First Medical Center of the Chinese People's Liberation Army General Hospital (ky‐2018‐3‐14), and all participants provided written informed consent. All the procedures involving human participants were performed in accordance with the World Medical Association Declaration of Helsinki (1964). This study was registered at ClinicalTrials.gov (NCT03613701).

### 2.3 Angiographic evaluation

Cerebral angiography was routinely performed within 1 week before surgery. Angiographic staging was performed according to the Suzuki staging system (Suzuki & Takaku, 1969). Angiographic collateral grading was performed as previously described, with a score range of 1−12 points (Liu et al., 2019). The anatomic extent of pial collateral blood flow during the venous delay period ranged from the area supplied by the posterior cerebral artery to the area supplied by the anterior cerebral artery and middle cerebral artery (MCA). Basal brain perforators and smoke vessels were determined according to Suzuki staging. The condition of collateral circulation in patients with MMD was divided into three stages: scores of 1−4 were defined as poor collateral circulation (stage I); scores of 5−8 were defined as good collateral circulation (stage II); and scores of 9−12 were defined as excellent collateral circulation (stage III).

All patients underwent a follow‐up angiography 6 months after EDAS. The development of collateral circulation in the MCA via the bypass was graded according to the grading system described by Matsushima et al. (1992): grade A, the blood supply area of the surgical bypass covered more than 2/3 of the MCA distribution; grade B, the area covered 1/3−2/3 of the MCA distribution; grade C, the area covered only 1/3 of the MCA distribution; and grade D, no collateral circulation was observed.

All angiographic images were reviewed by two experienced viewers (Qiannan Wang and Gan Gao) who were unaware of the angiographic results and clinical details. In case of disagreement, a consensus was reached after discussion.

### 2.4 Magnetic resonance imaging evaluation

One week before and 6 months after surgery, the cerebral hemodynamic status was assessed by dynamic susceptibility contrast magnetic resonance imaging (DSC‐MRI) using a MAGNETOM Skyra 3T MRI scanner (Siemens, Germany) following previously described methods (Qiao et al., 2017). The acquired DSC‐MR images were processed using a postprocessing workstation (Syngo Via 20, Siemens) and analyzed using the MR Neuro‐Perfusion software. We used the time to peak (TTP; the time at which the contrast level reached its maximum) and the mean relative cerebral blood volume (rCBV; a hemodynamic parameter calculated by MR perfusion) to evaluate the hemodynamic status of the patients.

### 2.5 Homocysteine assay

Blood samples were drawn from fasting patients and collected in tubes containing ethylenediaminetetraacetic acid. Plasma Hcy samples were placed on ice and transported to the laboratory within 30 min of collection, and the blood was centrifuged for 1−2 h after venipuncture. Plasma was frozen at −20°C until analysis. The Hcy concentration was determined using a competitive immunoassay (Colson et al., 2017; Fekih‐Mrissa et al., 2017).

### 2.6 Surgical procedure

All enrolled patients underwent EDAS performed by the same neurosurgeon. For specific details about the surgical methods, please refer to our previous studies (Bao et al., 2018; Wang et al., 2019).

### 2.7 Clinical follow‐up

Long‐term outcomes were ascertained through clinical visits and telephone calls. The clinical outcomes were divided into four grades: (1) excellent, in that the preoperative symptoms had completely disappeared with no fixed neurological deficits; (2) good, in that the symptoms had markedly decreased, but neurological deficits remained; (3) fair, in that the symptoms persisted less frequently; and (4) poor, in that the preoperative status remained either unchanged or worsened, or new symptoms appeared (Matsushima et al., 1989; Seol et al., 2005). The modified Rankin Scale (mRS) was used to determine the neurological functional outcomes before and after surgery (The Dutch T1A Study Group, 1988). Perioperative stroke was defined as the presence of either infarction or hemorrhage that developed during surgery or within 1 month after surgery on MR or CT tomography. Late postoperative stroke was defined as an event that occurred during the follow‐up period.

### 2.8 Statistical analysis

The baseline characteristic data of all patients are presented as the mean ± standard deviation (continuous variable) and *n* (%) (categorical variable). The χ^2^ test was used to compare categorical variables, and the independent sample *t*‐test or analysis of variance was used to compare continuous variables. Univariate and multivariate logistic regression analyses were performed to determine risk factors for the clinical outcomes. Differences were considered statistically significant at *p* < .05. All statistical analyses were performed using SPSS software for Windows (version 20.0; IBM).

## 3 RESULTS

### 3.1 Patient characteristics

A total of 123 patients were included in the study, including 41 patients in the HHcy group and 82 patients in the non‐HHcy group. Table 1 shows the baseline characteristics of the patients. The mean age at MMD onset was 39.0 ± 12.2 in the HHcy group and 38.4 ± 9.6 in the non‐HHcy group. In the HHcy group, 9 patients (22.0%) were female, which was significantly less than the number of female patients in the non‐HHcy group (52 cases, 63.4%) (*p* = .001). Transient ischemic attack (TIA) was the most common initial symptom in both groups. In the HHcy group, 13 patients (34.2%) smoked or consumed alcohol, which was significantly more than the corresponding number of 11 patients (13.9%) in the non‐HHcy group (*p* = .011). Posterior cerebral artery stenosis was found in 18 patients (43.9%) in the HHcy group and 42 patients (51.2%) in the non‐HHcy group (*p* = .444). There was no significant difference in mRS scores between the two groups.

**TABLE 1:** Clinical comparison of the HHcy and non‐HHcy groups.

Patient characteristics^a^	Total (*n* = 123)	HHcy group (*n* = 41)	Non‐HHcy group (*n* = 82)	*p* Value
Mean age ± SD, years	38.6 ± 10.5	39.0 ± 12.2	38.4 ± 9.6	.781
Female	61 (49.6%)	9 (22.0%)	52 (63.4%)	.001
Initial symptoms				
Asymptomatic	2 (1.6%)	1 (2.4%)	1 (1.2%)	.568
TIA	52	18 (43.9%)	35 (42.7%)	
Infarction	(43.1%)	5 (12.2%)	19 (23.2%)	
Hemorrhage	24 (19.5%)	10 (24.4%)	16 (19.5%)	
Others (headache, epilepsy)	26 (21.1%)	7 (17.1%)	11 (13.4%)	
Stroke risk factors	18 (14.6%)			
Hypertension	51	21 (51.2%)	30 (36.6%)	.120
Diabetes mellitus	(41.5%)	5 (12.2%)	19 (23.2%)	.148
Hyperlipidemia smoking or drinking	24 (19.5%)	19 (46.3%)	38 (46.3%)	1.000
		13 (34.2%)	11 (13.9%)	.011
	57 (46.3%)			
	24 (20.5%)			
PCI	60 (48.8%)	18 (43.9%)	42 (51.2%)	.444
Admission mRS score				
0	3 (2.4%)	1 (2.4%)	2 (2.4%)	.150
1	42	12 (29.3%)	30 (36.6%)	
2	(34.1%)	18 (43.9%)	28 (34.1%)	
3	46	4 (9.8%)	18 (22.0%)	
4	(37.4%) 22	6 (14.6%)	4 (4.9%)	
5	(17.9%)	0	0	
6	10 (8.1%)	0	0	
	0			
	0			

HHcy: hyperhomocysteinemia; TIA: transient ischemic attack; PCI: posterior circulation involvement; mRS: modified Rankin Scale.

^a^Values are presented as the number of patients (%) unless otherwise noted.

### 3.2 Angiography and magnetic resonance imaging outcomes

The mean follow‐up time was 17.38 ± 8.47 months (range: 6−48 months) in the HHcy group and 18.23 ± 9.35 months (range: 6−52 months) in the non‐HHcy group, with no significant difference. There was no significant difference in the preoperative Suzuki stage between the two groups (*p* = .187). We found further progression of peripheral Willis circle vascular lesions in both groups after surgery. However, there was no significant difference in the Suzuki stage between the two groups (*p* = .469) (Table 2).

**TABLE 2:** Preoperative and postoperative Suzuki stage between the two groups.

	Preoperative Suzuki stage			Postoperative Suzuki stage		
Suzuki stage^a^	HHcy group	Non‐HHcy group	*p* Value	HHcy group	Non‐HHcy group	*p* Value
1	12 (14.6%)	21 (12.8%)	.187	10 (12.2%)	21 (12.8%)	.469
2	20 (24.4%)	23 (14.0%)		15 (18.3%)	17 (10.4%)	
3	13 (15.9%)	25 (15.2%)		13 (15.9%)	22 (13.4%)	
4	18 (22.0%)	32 (19.5%)		20 (24.4%)	39 (23.8%)	
5	8 (9.8%)	27 (16.5%)		10 (12.2%)	25 (15.2%)	
6	11 (13.4%)	36 (22.0%)		14 (17.1%)	40 (24.4%)	
Total	82	164		82	164	

HHcy: hyperhomocysteinemia.

^a^Values are presented as the number of hemispheres (%). Both hemispheres were.

evaluated for all patients.

†*p* < .05 versus the non‐HHcy group.

Regarding angiographic collateral grading, there was no significant difference between the two groups in terms of collateral compensation of the pia, which was mainly low‐grade compensation. As determined by the Matsushima grades, patients in the non‐HHcy group were more likely to develop collateral neovascularization after surgery, showing a greater coverage of neovascularization with a significant difference (*p* = .002) (Table 3).

**TABLE 3:** Collateral grade, Matsushima grade, and DSC‐MRI on the operative hemisphere between the two groups.

Variables^a^	HHcy group	Non‐HHcy group	*p* Value
Collateral grade			
Poor (stage I)	71 (86.6%)	128 (78.0%)	.205
Fair (stage II)	8 (9.8%)	21 (12.8%)	
Good (stage III)	3 (3.7%)	15 (9.1%)	
Matsushima grade			
Poor	12 (14.6%)	14 (8.5%)	.002
Fair (< 1/3 of MCA)	20 (24.4%)	18 (11.0%)	
Good (2/3 to 1/3 of MCA)	42 (51.2%)	91 (55.5%)	
Excellent (> 2/3 of MCA)	8 (9.8%)	41 (25.0%)	
DSC‐MRI on preoperative			
TTP (s)	4.2 ± 14.2	3.8 ± 3.1	.760
rCBV (mL/100 g)	1.7 ± 0.7	1.9 ± 0.9	.184
DSC‐MRI on postoperative			
TTP (s)	2.4 ± 2.1	2.5 ± 1.9	.036
rCBV (mL/100 g)	1.6 ± 0.6	1.8 ± 0.7	.217

HHcy: hyperhomocysteinemia; MCA, middle cerebral artery; DSC‐MRI, dynamic susceptibility contrast magnetic resonance imaging; TTP, time to peak; rCBV, relative cerebral blood volume.

^a^Values are expressed as the number of operative hemispheres (%).

†*p* < .05 versus the non‐HHcy group.

In terms of hemodynamic status, there was no significant difference in the preoperative TTP and rCBV between the two groups. Postoperative review showed that the TTP and rCBV of the two groups significantly improved after surgery. The difference in TTP was significant (*p* = .036), whereas the difference in rCBV was not (*p* > .05) (Table 3).

### 3.3 Results of follow‐up

All 123 patients underwent bilateral EDAS surgery and received a timely follow‐up, with a median follow‐up period of 67.58 ± 6.19 months (range: 61−70 months). The mean follow‐up time was 65.78 ± 5.48 months (range: 60−71 months) in the HHcy group and 68.95 ± 6.47 months (range: 62−72 months) in the non‐HHcy group. The difference was not significant (*p* > .05). During the follow‐up, 10 patients had a late stroke and none died. Additionally, there were four cases of ischemic stroke and one case of hemorrhagic stroke in the HHcy group, as well as four cases of ischemic stroke and one case of hemorrhagic stroke case in the non‐HHcy group. The probability of postoperative stroke in the HHcy group was significantly higher than that in the non‐HHcy group (*p* < .05). In terms of clinical outcomes, 15 patients (36.6%) in the HHcy group had unchanged or worsened clinical symptoms after surgery, which was significantly more than the corresponding number of 6 patients (7.3%) in the non‐HHcy group. Meanwhile, six patients (14.6%) in the HHcy group were lost to follow‐up after surgery, which was significantly less than the corresponding number of 27 patients in the non‐HHcy group (32.9%) (*p* < .001) (Table 4).

**TABLE 4:** Distribution of clinical outcomes between the two groups.

Clinical outcome^a^	HHcy group	Non‐HHcy group	*p* Value
Poor	15 (36.6%)	6 (7.3%)	<.001
Fair	18 (43.9%)	14 (17.1%)	
Good	2 (4.9%)	35 (42.7%)	
Excellent	6 (14.6%)	27 (32.9%)	
Total	41	82	

HHcy: hyperhomocysteinemia.

^a^Values are presented as the number of patients (%).

†*p* < .05 versus the non‐HHcy group.

Most patients in both groups showed significant improvements in mRS during the postoperative follow‐up. However, significantly better mRS results were observed in the non‐HHcy group compared to the HHcy group (*p* = .020) (Table 5).

**TABLE 5:** . mRS assessment during follow‐up.

mRS Score^a^	HHcy group	Non‐HHcy group	*p* Value
0	6 (14.6%)	27 (32.9%)	.020
1	18 (43.9%)	40 (48.8%)	
2	6 (14.6%)	8 (9.8%)	
3	7 (17.1%)	6 (7.3%)	
4	4 (9.8%)	1 (1.2%)	
5	0	0	
6	0	0	
Total	41	82	

mRS: modified Rankin Scale; HHcy: hyperhomocysteinemia.

^a^Values are presented as number of patients (%).

†*p* < .05 versus the non‐HHcy group.

Univariate logistic regression analysis of preoperative clinical variables showed that an adverse postoperative clinical outcome of MMD EDAS was associated with HHcy (odds ratio [OR]: 7.31; 95% confidence interval [CI]: 2.57−20.81; *p* < .001), smoking or alcohol consumption (OR: 4.15; 95% CI: 1.42−12.13; *p* = .009), and late postoperative stroke (OR: 45.45; 95% CI: 8.81−234.54; *p* < .001) (Table 6). Multivariate logistic regression analysis of preoperative clinical variables identified HHcy (OR: 25.11; 95% CI: 2.95−213.67; *p* = .03), smoking or alcohol consumption (OR: 6.72; 95% CI: 1.12−40.17; *p* = .037), and late postoperative stroke (OR: 264.96; 95% CI: 15.11−4647.31; *p* < .001) as predictors of adverse clinical outcomes (Table 7).

**TABLE 6:** Univariate logistic regression analyses for predictive factors of clinical outcomes.

Variable	OR	95% CI	*p* Value
Age on set	1.03	0.98−1.07	.250
Sex	0.57	0.22−1.49	.251
Asymptomatic	0	0–Inf	.993
TIA	1.26	0.49−3.23	.637
Infarction	0.16	0.02−1.24	.079
Hemorrhage	1.18	0.39−3.58	.776
Others (headache, epilepsy)	2.29	0.71−7.39	.165
Hypertension	1.35	0.53−3.47	.530
Diabetes mellitus	1.87	0.64−5.47	.255
Hyperlipidemia	0.52	0.19−1.40	.194
Smoking or drinking	4.15	1.42−12.13	.009
Hyperhomocysteinemia	7.31	2.57−20.81	<.001
PCI	0.95	0.37−2.42	.907
Late postoperative stroke	45.45	8.81−234.54	<.001

OR: odds ratio; CI: confidence interval; TIA: transient ischemic attack; PCI: posterior circulation involvement.

**TABLE 7:** Multivariate logistic regression analyses for predictive factors of clinical outcomes.

Variable	OR	95% CI	*p* Value
Infarction	0.20	0.01−2.62	.218
Hemorrhage	0.26	0.04−1.65	.155
Smoking or drinking	6.72	1.12−40.17	.037
Hyperhomocysteinemia	25.11	2.95−213.67	.003
PCI	0.31	0.06−1.54	.150
Late postoperative stroke	264.96	15.11−4647.31	<.001

CI, confidence interval; PCI: posterior circulation involvement.

## 4 DISCUSSION

Indirect revascularization is one of the main surgical methods for treating MMD, with EDAS the most widely used among them. The success of EDAS is marked by postoperative collateral neovascularization. Although previous studies have reported factors affecting collateral angiogenesis (Kim et al., 2007; Ren et al., 2016; Sakamoto et al., 2007; Wang et al., 2021), few studies have investigated the effect of HHcy. However, the mechanism underlying collateral vascularization after EDAS remains unclear. Further studies are urgently needed to identify the factors that influence collateral vascularization after EDAS to improve the success rate of surgery in patients with MMD. In this retrospective study, we found that patients with MMD complicated by HHcy had poor collateral angiogenesis after EDAS, faster disease progression, and worse clinical outcomes. To the best of our knowledge, this is the first study to investigate the effect of HHcy on collateral angiogenesis after EDAS in patients with MMD.

Previous studies have shown that Hcy concentration is affected by the dietary intake of folic acid and B vitamins, smoking, alcohol consumption, sex, and other factors, as well as genetic factors (Colson et al., 2017; Frosst et al., 1995; Klerk et al., 2002). Thus, MTHFR polymorphisms, male sex, smoking, and alcohol consumption lead to elevated plasma Hcy levels (Castro et al., 2003; Chmurzynska et al., 2013; Li et al., 2021). In this study, the proportion of male patients was significantly higher than that of female patients. Simultaneously, the proportions of patients who smoked and consumed alcohol in the HHcy group were significantly higher than the corresponding proportions in the non‐HHcy group, which is consistent with the results of previous studies. Bao et al. (2010) and Chen et al. (2004) believed that HHcy was an independent risk factor for the development of cardiovascular and cerebrovascular diseases. Additionally, an increasing number of studies have demonstrated that HHcy is closely related to the risk of ischemic cardiovascular and cerebrovascular diseases (Anniwaer et al., 2019; Peng et al., 2020). The results of this study showed that the two groups of patients had different degrees of disease progression after EDAS, as indicated by Suzuki stages, compared to the preoperative stage. However, through statistical analysis, we found no significant difference in the preoperative and postoperative Suzuki stages between the two groups. This indicated that there was progression in both groups, but that HHcy was not a risk factor for MMD progression.

There are three accepted indicators for assessing the success of surgery in the treatment of MMD, the first of which is the Matsushima grade on cerebrovascular angiography. Our results showed that the Matsushima grade after EDAS of patients in the HHcy group was significantly worse than that of the patients in the non‐HHcy group, indicating that HHcy inhibited collateral neovascularization. Hcy has been suggested to induce peripheral vascular apoptosis in vitro by regulating mitochondrial dysfunction and autodevelopment of the MIF/mTOR signaling pathway (Fan et al., 2019; Wang et al., 2019). It is also believed that the key to good collateral vessel growth lies in the high content of growth factors and cytokines in the body, including vascular endothelial growth factor (VEGF) and basic fibroblast growth factor (Prakash et al., 2012; Weinberg et al., 2011). However, the mechanism by which HHcy inhibits collateral angiogenesis after EDAS remains unclear, and is the subject of the current study. The second accepted indicator is parameters observed during DSC‐MRI examination. We used TTP and rCBV to evaluate the hemodynamic status of patients. The results showed that the TTP and rCBV of patients in the two groups improved to varying degrees after EDAS surgery. However, the improvement in the TTP of patients in the non‐HHcy group was significantly greater than that in the HHcy group, indicating that cerebral blood flow was significantly improved in the non‐HHcy group after EDAS surgery. Finally, the clinical outcomes in patients were evaluated. In this study, we found that although most clinical symptoms improved in the two groups to varying degrees, the clinical outcomes in the non‐HHcy group were significantly superior to those in the HHcy group. Interestingly, during the follow‐up, we also found that the mRS scores of patients in the non‐HHcy group improved, which further indicated that HHcy affected the clinical outcomes of EDAS in patients with MMD. In the HHcy group, the clinical outcome of 15 patients showed that the preoperative status remained unchanged or worsened, and further analysis revealed that 14 of the 15 patients with MMD were not effectively controlled at postoperative review. Therefore, it is also a clinical problem that needs to be solved in which range of homocysteine level should be controlled for the best prognosis of rain in patients with MMD complicated with HHcy after surgery. Another study is being conducted on this topic and the conclusion will be published in the near future.

Previous studies have identified preoperative cerebral infarction, preoperative cerebral hemorrhage, high Suzuki stage, PCA, and postoperative delayed stroke as factors related to postoperative complications and clinical outcomes of MMD (Bao et al., 2015; Bao et al., 2012; Kim et al., 2005; Ren et al., 2016; Strother et al., 2014). Our univariate and multivariate logistic regression analyses further confirmed that smoking, alcohol consumption, and postoperative delayed stroke were associated with adverse clinical outcomes. Moreover, we found that HHcy was closely associated with adverse clinical outcomes after EDAS operation and can thus be used as a novel, specific predictor of adverse clinical outcomes after EDAS operation.

This study has some limitations. First, this was a single‐center, retrospective study, with a small number of patients with Hcy, calling for further studies to confirm the results. Patients without HHcy were matched according to age and sex, and other potential confounding factors could not be controlled. Second, we only examined the correlation between HHcy and indicators such as Matsushima grades and clinical outcomes after EDAS in patients with MMD. The lack of animal models for MMD may be the biggest obstacle in exploring the etiology, pathology, and pathophysiological mechanisms of collateral circulation in patients with HHcy and non‐HHcy MMD. Therefore, we were unable to conduct a bench study to explain these findings.

## 5 CONCLUSIONS

Patients with MMD complicated with HHcy showed less efficient collateral neovascularization after EDAS surgery. HHcy levels can be used as a specific predictor of adverse clinical outcomes in patients with MMD.

## CONFLICT OF INTEREST STATEMENT

The authors declare that there is no conflict of interest.

### ETHICS STATEMENT

This study was approved by the Research Ethics Committee of the First Medical Center of the Chinese People's Liberation Army General Hospital (ky‐2018‐3‐14).

### PATIENT CONSENT STATEMENT

All participants provided written informed consent prior to participating in the study.

### CLINICAL TRIAL REGISTRATION

This study was registered at ClinicalTrials.gov (NCT03613701).

### PEER REVIEW

The peer review history for this article is available at https://publons.com/publon/10.1002/brb3.3093.

## Data Availability

The data that support the findings of this study are available upon request from the corresponding author (Lian Duan).
